# Multiple begomoviruses found associated with cotton leaf curl disease in Pakistan in early 1990 are back in cultivated cotton

**DOI:** 10.1038/s41598-017-00727-2

**Published:** 2017-04-06

**Authors:** Muhammad Zubair, Syed Shan-e-Ali Zaidi, Sara Shakir, Muhammad Farooq, Imran Amin, Jodi A. Scheffler, Brian E. Scheffler, Shahid Mansoor

**Affiliations:** 1 National Institute for Biotechnology and Genetic Engineering, Faisalabad, Pakistan; 2 Pakistan Institute of Engineering and Applied Sciences, Nilore, Islamabad Pakistan; 3 Centre for Agricultural Biochemistry and Biotechnology, University of Agriculture, Faisalabad, Pakistan; 4 USDA-ARS, Crop Genetics Research Unit, 141 Experiment Station Rd, Stoneville, MS 38776 USA; 5 USDA-ARS, Genomics and Bioinformatics Research Unit, 141 Experiment Station Rd, Stoneville, MS 38776 USA

## Abstract

The first epidemic of cotton leaf curl disease (CLCuD) in early 1990’s in the Indian subcontinent was associated with several distinct begomoviruses along with a disease-specific betasatellite. Resistant cotton varieties were introduced in late 1990’s but soon resistance was broken and was associated with a single recombinant begomovirus named Burewala strain of *Cotton leaf curl Kokhran virus* that lacks a full complement of a gene encoding a transcription activator protein (TrAP). In order to understand the ongoing changes in CLCuD complex in Pakistan, CLCuD affected plants from cotton fields at Vehari were collected. Illumina sequencing was used to assess the diversity of CLCuD complex. At least three distinct begomoviruses characterized from the first epidemic; *Cotton leaf curl Multan virus*, *Cotton leaf curl Kokhran virus* and *Cotton leaf curl Alabad virus*, several distinct species of alphasatellites and cotton leaf curl Multan betasatellite were found associated with CLCuD. These viruses were also cloned and sequenced through Sanger sequencing to confirm the identity of the begomoviruses and that all clones possessed a full complement of the TrAP gene. A new strain of betasatellite was identified here and named CLCuMuB^Veh^. The implications of these findings in efforts to control CLCuD are discussed.

## Introduction

Cotton is the largest fiber producing and important cash crop in Pakistan and India^1^. Cotton leaf curl disease (CLCuD) is the major biotic constraint of cotton, transmitted by the insect vector *Bemisia tabaci* (whitefly)^2, 3^. CLCuD affects cotton crops across Pakistan and northwestern India causing severe yield losses^4^. Cotton plants affected by CLCuD exhibit specific symptoms such as upward and downward leaf curling, vein thickening and swelling, stunted growth and development of leaf-like enations. In Pakistan, CLCuD was observed for the first time on cotton near Multan in 1967. The disease spread widely and some more cotton varieties were affected by late 1970, but were not considered significant^5^. In 1989, the disease was noted on a newly released variety S12 grown at Kokhran near Multan and became epidemic, spreading to all the growing areas of Pakistan. The first epidemic of the disease was associated with the “Multan strain” of begomoviruses^6^. By introduction of resistant varieties developed through conventional breeding, cotton production in Pakistan was restored to pre-epidemic levels in the late 1990s^7^. Unfortunately, in 2001 it became evident that resistance was broken and symptoms of CLCuD were observed in all previously resistant varieties at Burewala, Pakistan^8^. This signaled a second epidemic of CLCuD which spread to all the cotton growing areas of Pakistan^8, 9^.

The CLCuD complex is caused by monopartite begomoviruses (genus *Begomovirus*, family *Geminiviridae*) transmitted by *B. tabaci* ^10^. Begomoviruses are of two types, bipartite and monopartite; bipartite consists of two genomic components DNA-A and DNA-B equal in size (~2.7 kb). Monopartite begomoviruses have a single genomic component similar to DNA-A of bipartite begomoviruses and are often associated with satellite molecules called betasatellite and alphasatellite. Betasatellite (~1.4 kb in size) is a pathogenicity determinant, encodes a single βC1 protein and depends on the helper virus for movement, replication and encapsidation^11^. Alphasatellites (~1.4 kb in size) encode their own replication associated (Rep) protein, replicate independently from their helper virus and are usually not required for pathogenicity^11^.

During the first epidemic, CLCuD was caused by begomovirus-betasatellite complex. CLCuD was associated with several distinct monopartite begomoviruses – *Cotton Leaf curl Multan virus* (CLCuMuV), *Cotton leaf curl Kokhran virus* (CLCuKoV), *Cotton leaf curl Alabad virus* (CLCuAV) and *Papaya leaf curl virus* (PaLCuV)^4, 6, 12^, and a single betasatellite (Cotton leaf curl Multan betasatellite [CLCuMuB])^13, 14^. Following the appearance of the second CLCuD epidemic in cotton, to present time, a predominant single recombinant begomovirus named as *Cotton leaf curl Kokhran virus*- Burewala (CLCuKoV-Bu), previously known as *Cotton leaf curl Burewala virus* (CLCuBuV), was associated with CLCuD in Pakistan and India^9^. However, the occasional occurrence of four other virus species and strains [CLCuKoV-Ko (2005), CLCuKoV-La (2008), CLCuKoV-Sha (2004, 2005) and CLCoMuV-Dar (2006)] was also observed on cotton in Pakistan (Table S3). CLCuKoV-Bu is a recombinant virus, consisting of sequences encoding complementary-sense genes derived from CLCuMuV and sequences encoding virion-sense genes and origin of replication derived fromCLCuKoV^9, 15^. The most interesting feature of CLCuKoV-Bu isolates, associated with resistance breaking, was the lack of a full-length transcriptional activator protein (TrAP) and a mutated C2 protein of only 35 amino acids (aa)^9^. Recently CLCuKoV-Bu, with a full complement TrAP, has also been identified showing severe symptoms of CLCuD^16^. The betasatellite associated with CLCuKoV-Bu was also found to be recombinant with most of its sequence from CLCuMuB, but also containing a small fragment of SCR region derived from tomato leaf curl betasatellite^16^.

The CLCuD complex is in a state of continuous change, evolving rapidly to overcome resistance by component capture, recombination and mutation^17^. Recently a bipartite begomovirus, *Tomato leaf curl New Delhi virus* (ToLCNDV) was identified associated with CLCuD in Pakistan^18, 19^. Interestingly, ToLCNDV isolated from cotton maintained a full complement of TrAP. Another study identified *Chickpea chlorotic dwarf* virus (CpCDV), a *Mastrevirus* in cotton showing leaf curl virus disease symptoms^20^. These results suggest that CLCuD complex has captured viruses that may have contributed to complete breakdown of resistance.

Here we have characterized begomoviruses and associated satellites from symptomatic samples of cotton collected in Vehari from lines that were being screened for virus resistance. In this study, we tried to understand the evolving nature and recent changes in begomovirus disease complexes by using rolling circle amplification (RCA) followed by next generation sequencing (NGS) and Sanger sequencing. Based on NGS and Sanger sequencing data, three distinct begomoviruses (CLCuMuV, CLCuKoV, CLCuAV) characterized from the first epidemic and several distinct alphasatellites and a single betasatellite species were identified associated with CLCuD. We also performed Southern blot hybridization for semi-quantification of begomoviruses and betasatellites. An important feature of the complex found in recent samples from Vehari is the absence of CLCuKoV-Bu in recent samples. Implications of these findings on begomovirus disease complexes are discussed.

## Material and Methods

### Sample collection, DNA extraction and rolling circle amplification

**Figure 1:**
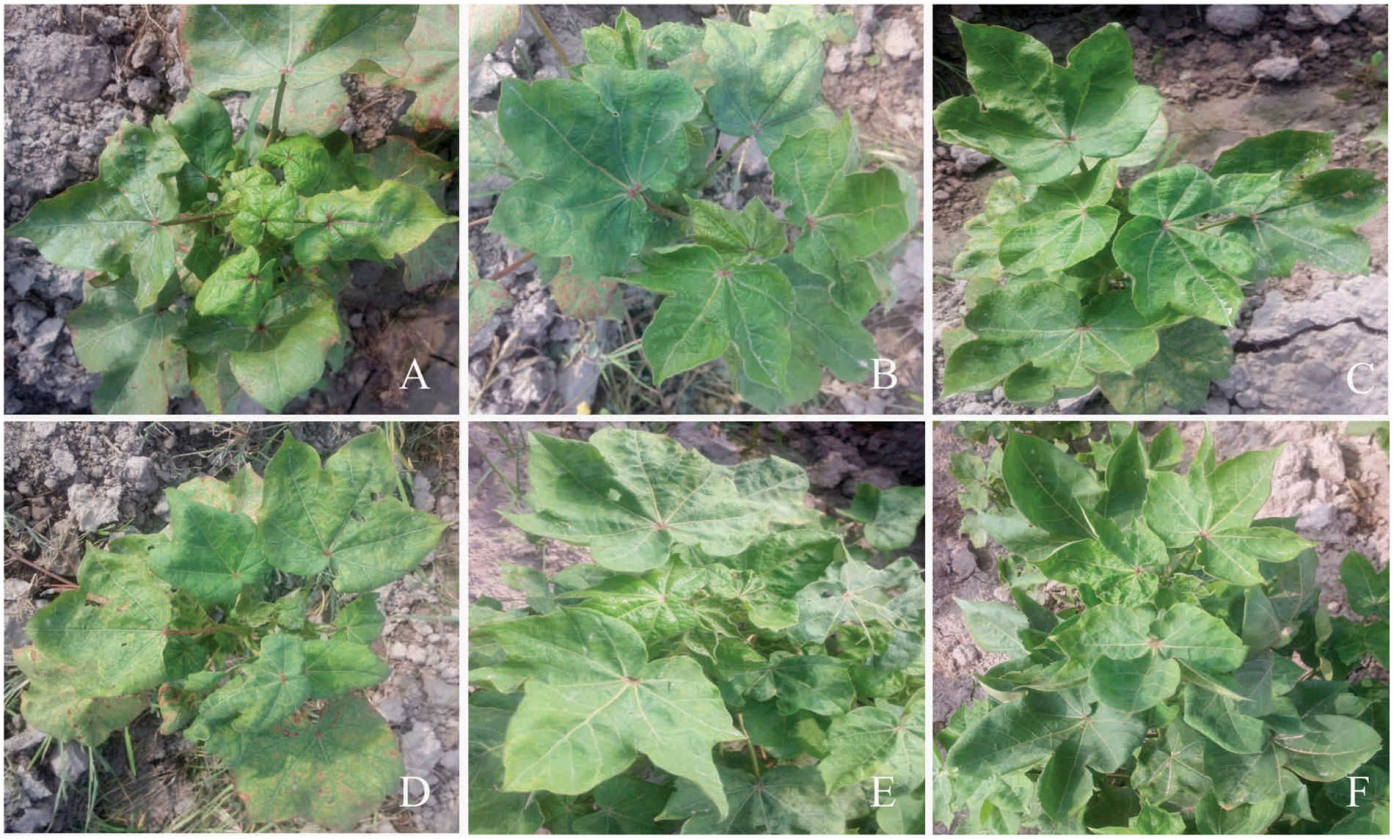
Cotton (*Gossypium hirsutum*) plants (**A–F**) from Vehari exhibiting typical symptoms of cotton leaf curl disease.

Cotton leaf samples from a total of six lines showing the typical CLCuD disease symptoms were collected from the Cotton Research Station (CRS) Vehari (Punjab province, Pakistan) in July 2015 (Fig. 1). Genomic DNA was extracted from infected samples using Cetyl trimethyl ammonium bromide (CTAB) method^21^, followed by ethanol precipitation and DNA quantification. To amplify circular molecules RCA^22^ was performed using phi 29 DNA polymerase (Thermo Fisher Scientific, Waltham, MA USA). RCA product was purified, enriched and processed for NGS.

### Library preparation and Illumina sequencing

The Illumina NeoPrep automation system (Illumina, San Diego, CA) was used with library kit, Illumina #NP-101-1001, “TruSeq Nano DNA Library Kit for NeoPrep”, which includes the adapter set “TruSeq LT”. The target insert size was 350 bp, with size selection performed by the NeoPrep instrument. Actual lower size limit of the libraries was ~300 bp as measured by the Agilent 2200 TapeStation. Sequencing was performed on the Illumina MiSeq, v2 chemistry, 2 × 150 bp.

### Nucleotide sequence assembly and analysis

The MiSeq Reporter software was set to automatically trim the adaptors. These short sequences were processed using CLC Genomics Work Bench 7.5. The paired-end reads obtained from the Illumina MiSeq Sequencer pipeline were subjected to quality filtering using quality score 0.001 and Phred quality score of 30. *De novo* as well as reference-guided assemblies were made. Reference-guided mapping was performed using begomovirus and satellite sequences present in Genbank. Based on good quality of data, twelve sequences from six plant samples (MW 6, MW 7, MW 8, MW 9, MW 10 and MW 11) were selected as shown in Table 1. All sequences were searched for similarity in NCBI non-redundant nucleotides database (nt), using BLASTn tool, provided by NCBI.

**Table 1:** Begomoviruses, betasatellites and alphasatellites obtained from infected cotton samples through NGS.

Sample	Sequence Name	Begomovirus/alpha/betas	Accession No	Size (nt)	No. of reads
MW6	MZ-50	CLCuMuV	KX603682	2738	1849
	MZ-51	CLCuAlV	KX656789	2737	1066
	MZ-77	CLCuMuB	KX656816	1340	4191
	MZ-78	CLCuMuB	KX656817	1410	1081
MW7	MZ-52	CLCuMuV	KX603683	2738	3916
	MZ-53	CLCuAlV	KX656790	2737	2517
	MZ-79	CLCuMuB	KX656818	1342	18760
	MZ-80	CLCuMuB	KX656819	1414	6982
	MZ-81	CLCuMuB	KX656820	1371	12405
	MZ-63	GDarSLA	KX656836	1372	3075
	MZ-64	GDarSLA	KX656837	1374	17579
	MZ-65	GDarSLA	KX656838	1370	1631
	MZ-67	CLCuMA	KX656839	1373	6371
MW8	MZ-54	CLCuMuV	KX603681	2738	1442
	MZ-55	CLCuAlV	KX656791	2737	2517
	MZ-82	CLCuMuB	KX656821	1340	4890
	MZ-83	CLCuMuB	KX656822	1358	3804
MW9	MZ-56	CLCuMuV	KX656786	2738	4776
	MZ-57	CLCuAlV	KX656792	2737	2981
	MZ-84	CLCuMuB	KX656823	1356	22044
	MZ-85	CLCuMuB	KX656824	1348	18284
	MZ-86	CLCuMuB	KX656825	1413	6082
MW10	MZ-58	CLCuMuV	KX656787	2738	2256
	MZ-59	CLCuAlV	KX656793	2737	1300
	MZ-87	CLCuMuB	KX656825	1413	6082
	MZ-88	CLCuMuB	KX656826	1370	1553
	MZ-68	GDarSLA	KX656840	1372	653
	MZ-69	GDavSLA	KX656847	1223	361
	MZ-71	GDavSLA	KX656848	1224	108
	MZ-73	GDavSLA	KX656849	1224	137
MW11	MZ-60	CLCuMuV	KX656788	2738	3895
	MZ-61	CLCuAlV	KX656794	2737	2299
	MZ-91	CLCuMuB	KX656828	1416	5303
	MZ-92	CLCuMuB	KX656829	1356	8829
	MZ-74	GDarSLA	KX656841	1372	2541
	MZ-76	GDavSLA	KX656842	1363	761
**Begomoviruses obtained through NGS with low percent coverage**					
Sample	Sequence Name	Begomoviruses	% age coverage	Size (nt)	No. of reads
MW6	MZ-100	CLCuKoV-Ko	50	2750	1131
	MZ-101	CLCuMuV-Raj	43	2740	114
MW7	MZ-102	CLCuKoV-Ko	55	2748	2277
	MZ-103	CLCuMuV-Raj	56	2738	237
MW8	MZ-104	CLCuKoV-Ko	50	2748	2816
	MZ-105	CLCuMuV-Raj	49	2736	212
MW9	MZ-106	CLCuKoV-Ko	60	2750	2816
	MZ-107	CLCuMuV-Raj	64	2738	356
MW10	MZ-108	CLCuKoV-Ko	51	2748	1397
	MZ-109	CLCuMuV-Raj	49	2738	137
MW11	MZ-110	CLCuKoV-Ko	56	2748	2350
	MZ-111	CLCuMuV-Raj	53	2736	210

### PCR amplification, cloning and Sanger sequencing of begomoviruses, alphasatellites and betasatellites

To amplify the begomovirus-complex from genomic DNA, primer pairs Begom-F/Begom-R^23^, Beta01/Beta02^24^ and DNA 101/DNA 102^25^ were used. For amplification of NGS-assembled CLCuAlV new sets of primers were designed (Table S4). The PCR products of ~2.8 kb, ~1.4 kb partial fragments for virus, and ~1.4 kb for alphasatellite and betasatellite, were cloned in a TA cloning vector ((pTZ57R/T; Thermo Fisher Scientific, Waltham, MA USA). An average of five clones per plants were selected for sequencing. Plasmids of the desired clones were purified using AxyPrep™ plasmid miniprep kit (Axygen, USA) and sequenced commercially on an Applied Biosystems 3730XL DNA sequencer (USA). The sequences were assembled and analyzed using Lasergene software (DNAStar Inc., Madison, USA). All the sequences were analyzed and compared to the sequences available in the data bank (NCBI) using BLASTn search tool.

### SDT and phylogenetic analysis

Sequence demarcation tool (SDT) v1.2^26^ was used for identification of species and strains of begomoviruses based on muscle alignment and identity score matrixes. Full length viruses and satellites sequences were aligned using pairwise multiple MUSCLE alignment algorithm in MEGA6^27^. This alignment was used to construct phylogenetic trees supported with 1000 bootstrap values through a neighbor-joining algorithm. Phylogenetic trees were edited and labelled in MEGA 6.

### Recombination analysis

Recombination among viruses and satellites were detected using RDP4 Beta 4.74^28^. RDP using 9 recombination analysis methods, RDP^29^, GENECONV^30^, BOOTSCAN^31^, MAXIMUM CHI SQUARE^32^, CHIMAERA^33^, SISCAN^34^, 3SEQ^35^, PHYLPRO^36^, LARD^37^ and VisRD^38^ for possible recombination breakpoints within query sequences. Recombination events identified by multiple recombination methods with high p-values and good phylogeny were considered potential targets for recombination.

### Southern blot hybridization

For Southern blot hybridization an agarose gel (1.2% [w/v]) stained in ethidium bromide was cast in a tray and an equal amount of DNA (~10 µg) per sample was resolved on the gel. After washing in depurination (0.25 M HCl), denaturation (1.5 M NaCl and 0.5 M NaOH) and neutralization buffer [1 M Tris (pH 7.4) & 1.5 M NaOH] the gel was blotted on Hybond-N^+^ membrane (GE Healthcare, UK) and UV cross-linked. The blot was pre-hybridized at 42 °C for 2–3 h in DIG Easy Hyb solution (Roche, Germany) and hybridized overnight at 42 °C in DIG-labelled probe. For begomoviruses, primers CLCV1/CLCV2. (5′-CCGTGCTGCTGCCCCCATTGTCCGCGTCAC-3′/5′-CTGCCACAACCATGGATTCACGCACAGGG-3′) and for betasatellites Beta01/Beta02 (5′-GTACCGGCTGCTGCGTAGCGTAGT-3′/5′-GGTACCTACCCTCCCAGGGGTACAC-3′) primers were used for synthesis of probes. Genomic DNA of infected cotton plants was used as insert to amplify DIG labeled probes. Hybridization signals were detected on blot after treatment with Nitro blue tetrazolium chloride (NBT) and 5-bromo-4-chloro-3-indolyl phosphate [(BCIP, Thermo Scientific™, USA)].

## Results

### Begomoviruses, alphasatellites and betasatellites obtained through NGS

The *de novo* assembly of the high-throughput Illumina sequencing data was carried out on CLC Genomics Work bench 7.5 (CLC bio) software. Resulting contigs of size ~2.8 kb and 1.4 kb, based on putative genome size of begomoviruses and alpha/betasatellites respectively, were selected from the data. The selected contigs were searched by using the BLASTn search tool for closely related begomoviruses and satellites molecules available at NCBI-GenBank database. From the NGS data, BLASTn indicated three kinds of begomoviruses, *Cotton leaf curl Multan virus* ***-***Pakistan (CLCuMuV-PK), *Cotton leaf curl Alabad virus* ***-***Multan (CLCuAlV-Mu) and *cotton leaf curl Kokhran virus*-Kokhran (CLCuKoV-Ko) were present. However, CLCuKoV-Ko sequences identified here with NGS have low percent (50–60%) coverage as shown in Table 1.

The BLASTn searches for satellite molecules identified three types of alphasatellites, *Gossypium darwinii* symptomless alphasatellite (GDarSLA), *Gossypium davidsonii* symptomless alphasatellite (GDavSLA) and cotton leaf curl Burewala alphasatellite (CLCuBuA) and one type of betasatellite, cotton leaf curl Multan betasatellite (CLCuMuB) in the collected infected samples (Table 1).

### Analysis and ORF identification of begomovirus, alphasatellites and betasatellites obtained through Sanger sequencing

The 2.8 kb and 1.4 kb clones obtained here were checked for the presence of putative open reading frames (ORF) using ORF finder at (www.ncbi.nlm.nih.gov/gorf). The ORF analysis shows that 2.8 kb clones resembled and had an arrangement of genes typical of the DNA-A component of begomoviruses (Table 2). Similarly, 1.4 kb clones resembled to alpha/betasatellites molecules respectively. The BLASTn and SDT results of Sanger sequencing data showed that 2 strains of Multan virus, CLCuMuV-PK and *Cotton leaf curl Multan virus*-Rajasthan (CLCuMuV-Raj) and one strain of CLCuKoV, *Cotton leaf curl Kokhran virus*-Shadadpur (CLCuKoV-Sha) were identified (Table 2). However, CLCuAlV identified with NGS was not amplified by universal begomo primers. Primer sets (Table S4) were designed according to the assembled sequences of NGS for detection of CLCuAlV in the diseased samples. PCR with the new sets of primers confirmed the presence of CLCuAlV in the diseased samples.

**Table 2:** Features of begomoviruses obtained from infected cotton samples through Sanger sequencing.

Sample	Clone Name	Virus Component	Location (province/District	Accession no.	Size (nt)	Coding sequences [coordinates/no. of amino acids]					
						CP	V2	Rep	TrAP	REn	C4
MW6	MZ-4	CLCuMuV	Punjab/Vehari	KX656795	2739	276–1046/256	116–481/121	1495–2583/362	1146–1598/150	1049–1453/134	2127–2429/100
	MZ-1	CLCuMuV-Raj	Punjab/Vehari	KX656814	2737	274–1044/256	114–470/118	1495–2583/362	1146–1598/150	1049–1453/134	2127–2429/100
	MZ-2	CLCuMuV-Raj	Punjab/Vehari	KX656805	2737	274–1044/256	114–470/118	1495–2583/362	1146–1598/150	1049–1453/134	2127–2429/100
MW7	MZ-7	CLCuMuV-Raj	Punjab/Vehari	KX656806	2737	274–1044/256	114–470/118	1495–2583/362	1146–1598/150	1049–1453/134	2127–2429/100
	MZ-5	CLCuMuV	Punjab/Vehari	KX656796	2739	276–1046/256	116–481/121	1495–2583/362	1146–1598/150	1049–1453/134	2127–2429/100
	MZ-9	CLCuMuV-Raj	Punjab/Vehari	KX656807	2737	274–1044/256	114–470/118	1495–2583/362	1146–1598/150	1049–1453/134	2127–2429/100
	MZ-11	CLCuKoV-Sha	Punjab/Vehari	KX656802	2748	292–1062/256	132–488/118	1505–2593/362	1156–1608/150	1059–1463/134	2137–2439/100
MW8	MZ-15	CLCuMuV	Punjab/Vehari	KX656797	2739	276–1046/256	116–481/121	1495–2583/362	1146–1598/150	1049–1453/134	2127–2429/100
	MZ-10	CLCuMuV-Raj	Punjab/Vehari	KX656808	2737	274–1044/256	114–470/118	1495–2583/362	1146–1598/150	1049–1453/134	2127–2429/100
	MZ-19	CLCuKoV-Sha	Punjab/Vehari	KX656803	2748	292–1062/256	132–488/118	1505–2593/362	1156–1608/150	1059–1463/134	2137–2439/100
MW9	MZ-17	CLCuMuV	Punjab/Vehari	KX656799	2739	276–1046/256	116–481/121	1495–2583/362	1146–1598/150	1049–1453/134	2127–2429/100
	MZ-13	CLCuMuV-Raj	Punjab/Vehari	KX656809	2737	274–1044/256	114–470/118	1495–2583/362	1146–1598/150	1049–1453/134	2127–2429/100
	MZ-14	CLCuMuV-Raj	Punjab/Vehari	KX656810	2737	274–1044/256	114–470/118	1495–2583/362	1146–1598/150	1049–1453/134	2127–2429/100
	MZ-25	CLCuKoV-Sha	Punjab/Vehari	KX656804	2748	292–1062/256	132–488/118	1505–2593/362	1156–1608/150	1059–1463/134	2137–2439/100
MW10	MZ-20	CLCuMuV	Punjab/Vehari	KX656899	2739	276–1046/256	116–481/121	1495–2583/362	1146–1598/150	1049–1453/134	2127–2429/100
	MZ-24	CLCuMuV	Punjab/Vehari	KX656800	2739	276–1046/256	116–481/121	1495–2583/362	1146–1598/150	1049–1453/134	2127–2429/100
	MZ-21	CLCuMuV-Raj	Punjab/Vehari	KX656811	2737	274–1044/256	114–470/118	1495–2583/362	1146–1598/150	1049–1453/134	2127–2429/100
MW11	MZ-29	CLCuMuV	Punjab/Vehari	KX656801	2739	276–1046/256	116–481/121	1495–2583/362	1146–1598/150	1049–1453/134	2127–2429/100
	MZ-22	CLCuMuV-Raj	Punjab/Vehari	KX656812	2737	274–1044/256	114–470/118	1495–2583/362	1146–1598/150	1049–1453/134	2127–2429/100
	MZ-28	CLCuMuV-Raj	Punjab/Vehari	KX656813	2737	274–1044/256	114–470/118	1495–2583/362	1146–1598/150	1049–1453/134	2127–2429/100

Analysis of CLCuMuV-PK isolates showed that they have 92–96%, CLCuMuV-Raj isolates have 96–99% and CLCuKoV-Sha isolates have 96–98% sequence identity with sequences of their respective viruses available in the NCBI database (Figure S1A–C). Three types of alphasatellites, GDarSLA, CLCuBuA and Ageratum conyzoides symptomless alphasatellite (AConSLA) and one type of betasatellite, CLCuMuB were also identified from Sanger sequencing as shown in (Table 3).

**Table 3:** Features of betasatellites and alphsatellites obtained through Sanger sequencing.

Betasatellites obtained through Sanger sequencing						
Clone	Satellite/Original/Recombinant	Location (province/district)	Accession No	Size (nt)	[Coordinates/no. of amino acids]	
					βC1	Rep
MZ-32	CLCuMuB/NA	Punjab/Vehari	KX697597	1356	194–550/118	—
MZ-33	CLCuMuB/Original	Punjab/Vehari	KX697598	1369	194–550/118	—
MZ-34	CLCuMuB/Original	Punjab/Vehari	KX697599	1374	194–550/118	—
MZ-35	CLCuMuB/Recombinant	Punjab/Vehari	KX697600	1370	194–550/118	—
MZ-36	CLCuMuB/Original	Punjab/Vehari	KX697601	1410	194–550/118	—
MZ-37	CLCuMuB/Original	Punjab/Vehari	KX697602	1358	194–550/118	—
**Alphasatellites obtained through Sanger sequencing**						
MZ-38	CLCuBuA	Punjab/Vehari	KX656851	1394	—	77–1024/315
MZ-40	AConSLA	Punjab/Vehari	KX656850	1361	—	82–1029/315
MZ-41	GDarSLA	Punjab/Vehari	KX656852	1375	—	70–1017/315

The BLASTn searches for satellite molecules identified three types of alphasatellites, *Gossypium darwinii* symptomless alphasatellite (GDarSLA), *Gossypium davidsonii* symptomless alphasatellite (GDavSLA) and cotton leaf curl Burewala alphasatellite (CLCuBuA) and one type betasatellite, cotton leaf curl Multan betasatellite (CLCuMuB) in the collected infected samples (Table 2).

### Analysis of TrAP gene of begomoviruses

A detailed analysis of TrAP encoding gene showed that isolates, CLCuMuV-PK, CLCuMuV-Raj and CLCuKoV-Sha obtained here through Sanger sequencing encoded a putatively full-length TrAP of 150 amino acids as shown in Table 2.

### Recombination analysis of begomoviruses

**Figure 2:**
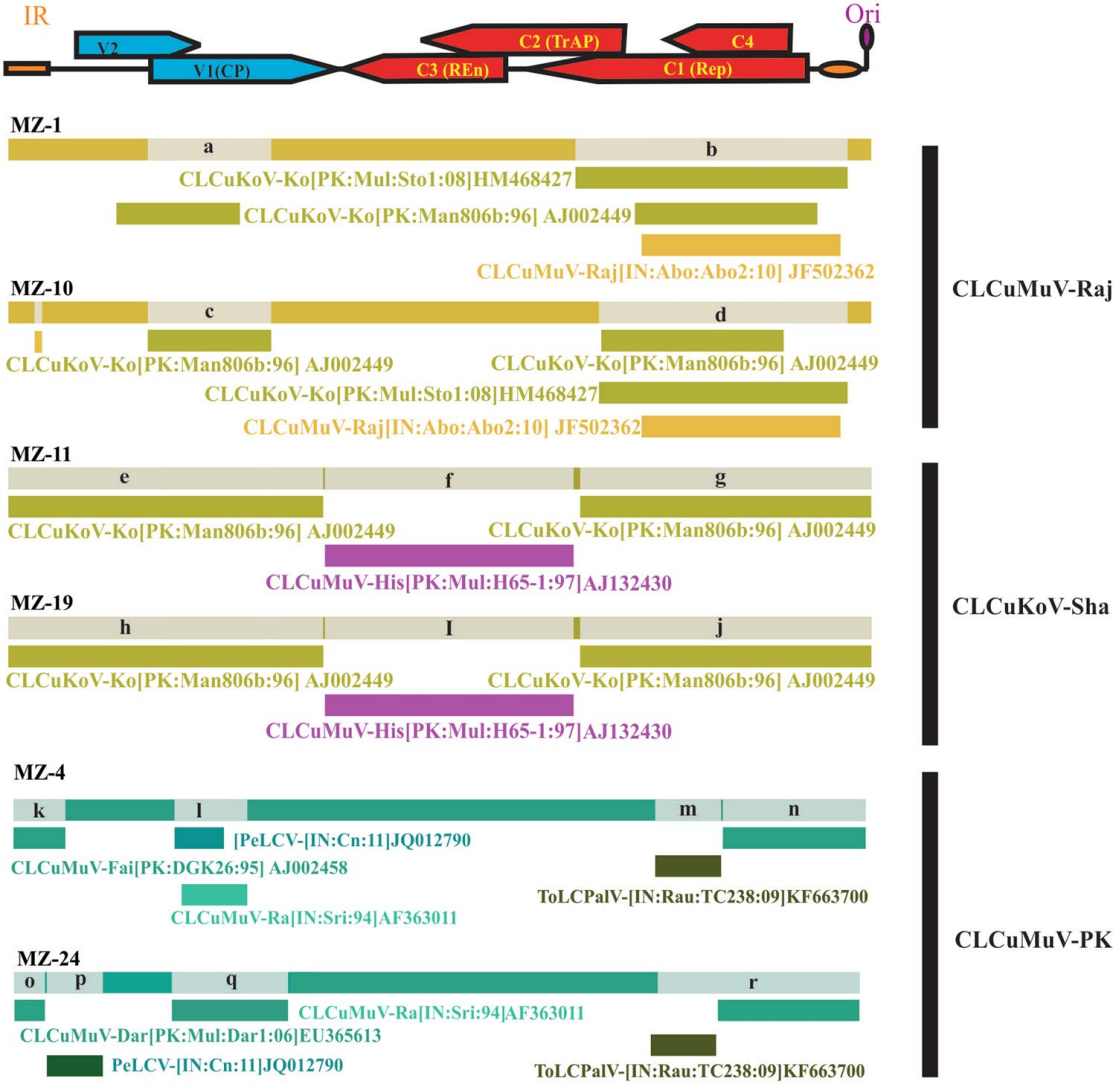
Recombination analyses of begomoviruses with RDP-4. Recombination analysis of *Cotton leaf curl Multan virus*-Rajasthan (CLCuMuV-Raj), *Cotton leaf curl Kokhran virus*-Shadadpur (CLCuKoV-Sha) and *Cotton leaf curl Multan virus*-Pakistan (CLCuMuV-PK). The isolates are grouped into species (marked on the right). A linear genome map of begomoviruses is plotted on top of figure, with position of genes and their orientation indicated by arrows to show recombinant fragments.

To determine recombination among begomoviruses isolates identified here, RDP4 Beta 4.74^28^ analysis was conducted. The isolates of CLCuMuV-Raj, CLCuKoV-Sha and CLCuMuV identified here were aligned with 260 full genome sequences of begomoviruses available and retrieved from the database (Table S1). The RDP result for begomoviruses is shown in Fig. 2 and further details including p-values are provided in Table S2.

Recombination analysis of CLCuMuV-Raj isolates show that CLCuMuV-Raj is a recombinant of two viruses CLCuMuV and CLCuKoV-Ko as reported previously^40^. Recombination analysis shows that CLCuKoV-Sha isolates contain recombinant regions of CLCuKoV-Ko in both virion-sense and complementary-sense strands. The CLCuMuV isolates contain a small fragment of CLCuMuV-Fai[PK:DGK26:95] and CLCuMuV-Raj[IN:Sri:94] in the coat protein region. The CLCuMuV isolates also contain fragments of *Pedilanthus leaf curl virus* (PeLCV-[IN:Cn:11]) in the coat protein and *Tomato leaf curl Palampur virus* (ToLCPalV-[IN:Rau:TC238:09]) in Rep in the complementary sense.

### Phylogenetic analysis of begomoviruses

**Figure 3:**
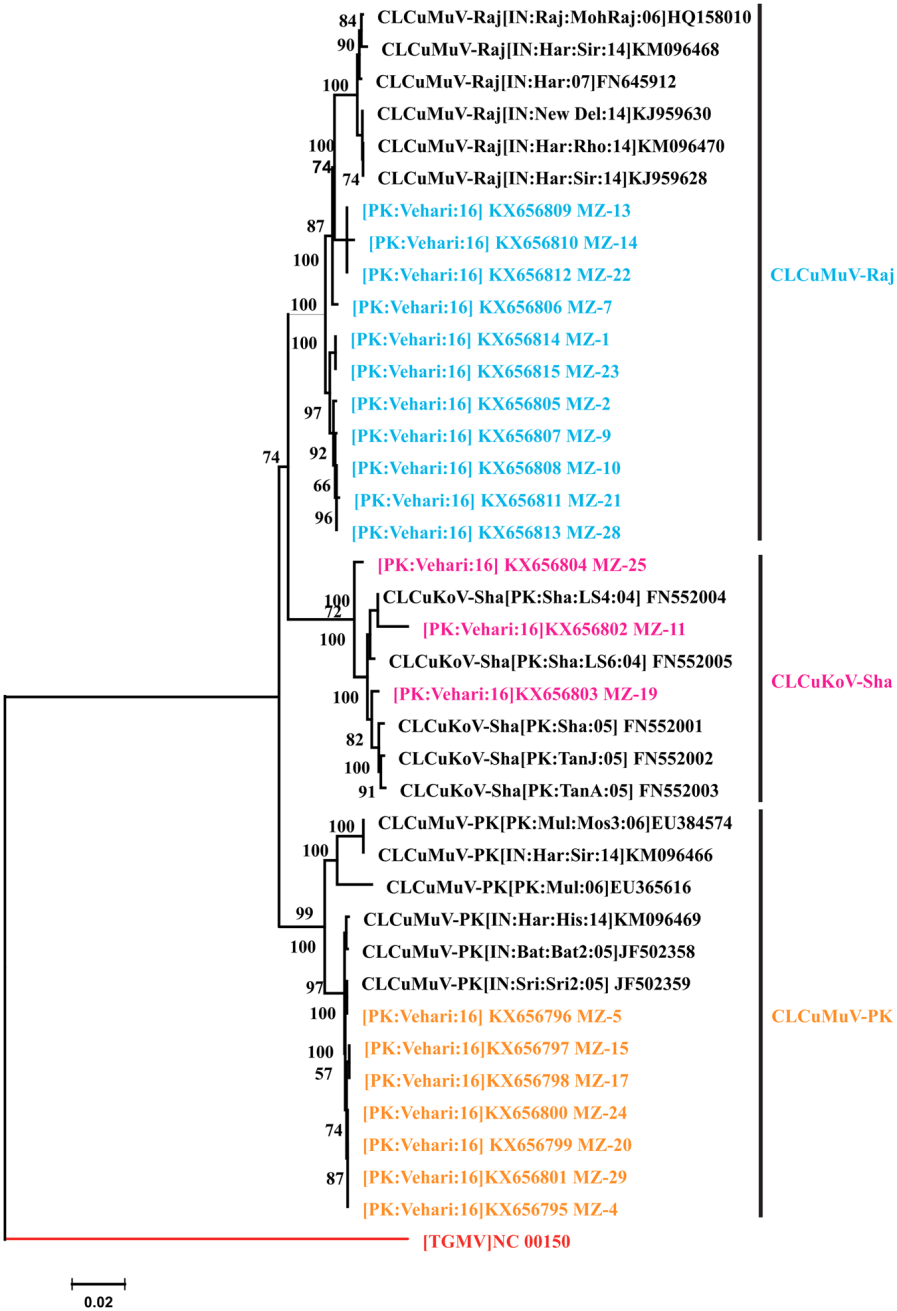
Phylogenetic tree of monopartite begomoviruses and DNA-A component. *Cotton leaf curl Multan virus*-Rajasthan (CLCuMuV-Raj) is colored sky blue, and *Cotton leaf curl Kokhran virus*-Shadadpur (CLCuKoV-Sha) is colored magenta and *Cotton leaf curl Multan virus*-Pakistan (CLCuMuV-P﻿K) is colored orange. The tree arbitrarily rooted on *Tomato golden mosaic virus* (ToMoV) was taken as outgroup is colored red. The tree was generated with 1000 bootstrap value represented along each root.

A phylogenetic dendrogram of begomoviruses was constructed on the basis of pairwise multiple MUSCLE alignment (MEGA6) of CLCuMuV, CLCuMuV-Raj and CLCuKoV-Sha and their similar isolates available in the database. The phylogenetic tree of CLCuMuV, CLCuMuV-Raj and CLCuKoV-Sha isolates shows that they are separated with closely related begomoviruses interspersed with the three begomoviruses as shown in Fig. 3.

### Analysis of alphasatellites

**Figure 4:**
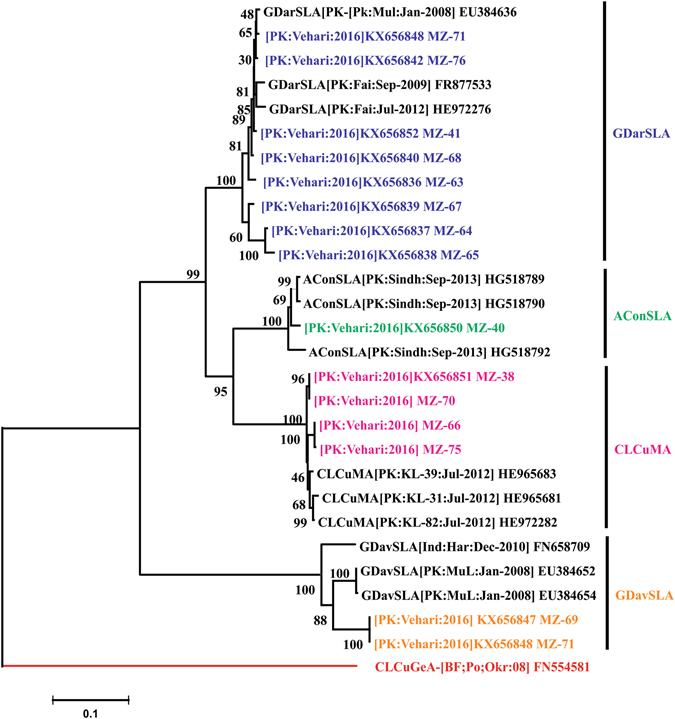
Phylogenetic tree of alphasatellites. *Gossypium darwinii* symptomless alphasatellite (GDarSLA) is colored orange, Ageratum conyzoides symtomless alphasatellite (AConSLA) is colored magneta and cotton leaf curl Burewala alphasatellite (CLCuBuA) is colored sky-blue. Cotton leaf curl Gezira alphasatellite (CLCuGeA) is taken as outgroup and colored red. The tree is supported by 1000 bootstrap value.

Three types of alphasatellites GDarSLA, CLCuBuA and AConSLA were identified from Sanger sequencing data. The phylogenetic tree of alphasatellites indicated that they clustered with closely related GDarSLA, CLCuBuA and AConSLA as shown in Fig. 4. RDP analysis of alphasatellites shows that sequence (MZ-40) consists of recombinant fragments of CLCuBuA and Croton yellow vein mosaic alphasatellite (CYVMA) sequences. RDP analysis of alphasatellites is shown with detail in Figure S2.

### Betasatsallites identified and analysis of SCR region

**Figure 5:**
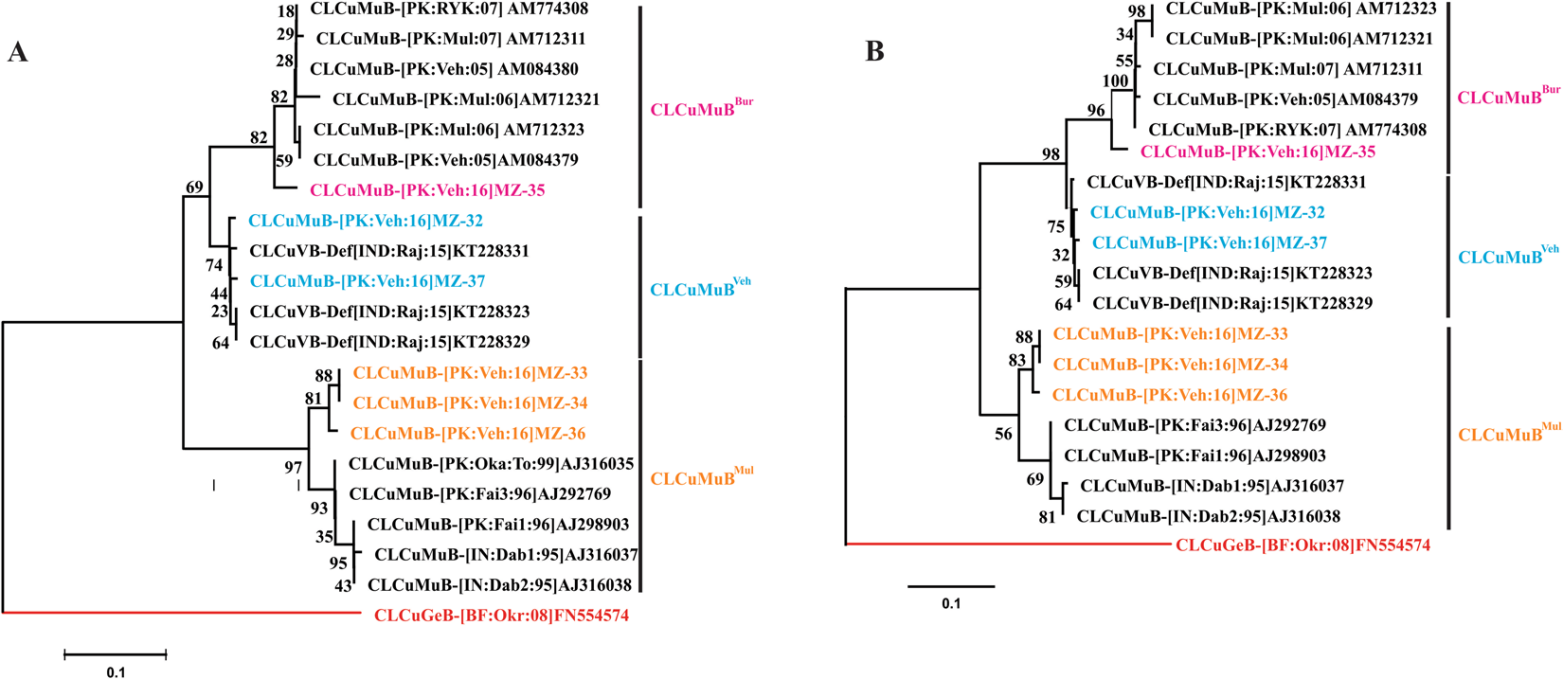
Phylogenetic tree of full length and SCR of betasatellite clones from cotton. **(A)** Phylogenetic tree of full length betasatellites. Cotton leaf curl Gezira betasatellite (CLCuGeB) was taken as outgroup. The tree is supported by 1000 bootstrap value. (**B)** Phylogenetic tree of SCR region of betasatellites. Three types of betasatellites were identified from both full and SCR alignments.

A single type of betasatellite, CLCuMuB was obtained from Sanger sequencing data. Analysis of the SCR region of these betasatellites showed that three types of betasatellites separated into three groups as shown in Fig. 5. The first group of betasatellites consisting of 3 clones (MZ-33, MZ-34, MZ-36) segregated with CLCuMuB which was associated with CLCuD in Pakistan during the 1990s. It was first identified by Briddon *et al*.^41^, and known as Multan betasatellite (CLCuMuB^Mul^). The second group consisted of a single clone (MZ-35) and is closely related to recombinant CLCuMuB associated with CLCuBuV known as (CLCuMuB^Bur^). The clone (MZ-35) contains approximately 95 nt derived from Tomato leaf curl betasatellite (ToLCuB) within the SCR region^17, 42^. The third group consisted of two clones (MZ-32, MZ-37) and their sequence in SCR does not match either CLCuMuB^Mul^ or ToLCuB. Instead they contain approximately 90 nt within the SCR derived from cotton leaf curl virus betasatellite defective interfering DNA (KT228331), recently identified from India, and here we designate it as Vehari betasatellite (CLCuMuB^Veh^). Phylogenetic trees of full length betasatellites and their SCR regions is shown in Fig. 5.

### Southern blot hybridization of begomoviruses and betasatellites

**Figure 6:**
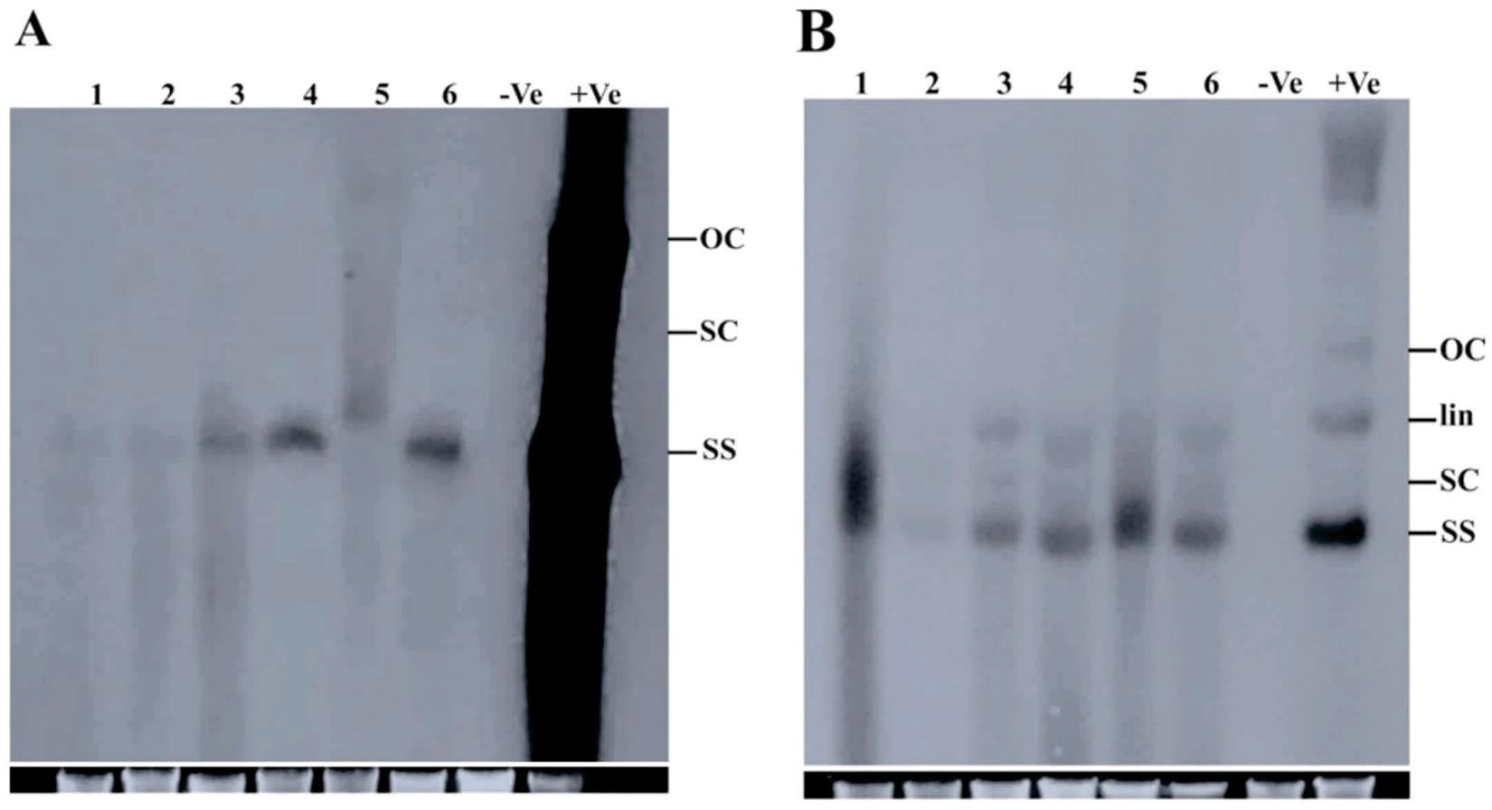
Southern blot hybridization analysis of begomovirus and betasatellite. (**A**) Southern blot hybridization with begomovirus probe. Genomic DNA from cotton samples were loaded in lane 1–6 respectively. Agro-infiltrated plants genomic DNA was used as positive control and healthy plant genomic DNA as negative control represented as +Ve and −Ve, loaded in lane 7 and 8 respectively. (**B**) Southern blot with betasatellite probe. Genomic DNA from cotton samples were loaded in lane 1–6 respectively. Positive control and negative control represented as +Ve and −Ve, loaded in lane 7 and 8 respectively. The different replicative forms of viral DNA are shown as open circular (oc), linear (lin), supercoiled (sc) and single stranded (ss).

To determine relative titer of begomoviruses and betasatellites, Southern blot hybridization of the infected cultivated cotton samples was performed. Southern blot analysis of DNA samples extracted from symptomatic leaves showed weak hybridization signals for virus probe in sample 1 and 2 indicating that virus titer was low in these samples, while virus titer was high in samples 3, 4, 5 and 6 showing good hybridization signals for virus probe as shown in (Fig. 6A). The titer of betasatellites was high in all the samples showing good hybridization signals with beta probe (Fig. 6B).

## Discussion

The begomovirus disease complex can severely affect yields of cultivated cotton in Pakistan and India, and previously often occurred as an infection of multiple begomoviruses. The CLCuKoV-Bu strain, with a truncated TrAP, is the only begomovirus strain associated with the resistance breaking of CLCuD currently found throughout Pakistan, with the occasional identification of other strains (Table S3)^9, 43^. We have found that some recent isolates of CLCuKoV-Bu encode a full length TrAP (Hassan *et al*., unpublished data). Our current study shows that the CLCuD complex can occur as result of infection of multiple begomoviruses. The begomovirus disease complex can easily evolve due to component capture, recombination and mutation in order to overcome disease resistance and to expand its host range^17, 44^.

To understand ongoing changes in the CLCuD complex, symptomatic leaves of previously resistant cotton varieties were collected from Vehari. Vehari is a major cotton growing area of Pakistan and exhibits a high diversity of begomoviruses. To evaluate the samples collected, a highly sensitive method of RCA coupled with NGS was used followed by confirmation with Sanger sequencing. Our NGS data showed that the three distinct begomoviruses, CLCuMuV, CLCuAlV and CLCuKoV-Ko associated with the first epidemic of CLCuD in early 1990s, a betasatellite, CLCuMuB and 3 alphasatellites GDarSLA, GDavSLA and CLCuBuA were identified in infected samples from Vehari. The reason for the low coverage (50–60%) of CLCuKoV-Ko in NGS data is not known. To confirm our NGS data results, genomic DNA from the collected samples were amplified with universal begomo, alpha and beta primers, cloned and dideoxy nucleotide chain termination sequencing was performed. From the Sanger sequencing data, two strains of Multan, CLCuMuV-Mu, CLCuMuV-Raj and one strain of Kokhran, CLCuKoV-Sha associated with the first epidemic of CLCuD, were identified. Our results from both NGS and Sanger sequencing data shows that CLCuKoV-Bu was not identified in infected cotton samples from Vehari. The presence of previously identified begomoviruses and the absence of CLCuKoV-Bu which was the only begomovirus found in infected cotton plants, indicate a potential major change in the begomovirus complex associated with CLCuD in Pakistan. A single species of betasatellite (CLCuMuB) and three species of alphasatellites (GDarSLA, CLCuBuA and AConSLA) were identified in these samples. Sanger sequencing data complementing our NGS data, identified and confirmed the presence of CLCuMuV, CLCuMuV-Raj and CLCuKoV-Sha. However, CLCuAlV identified with NGS was not amplified with universal begomo primers. The CLCuAlV was amplified from infected samples with CLCuAlV specific primers designed according to the assembled sequences of NGS (Table S4).

A closer detailed analysis of the TrAP gene of CLCuMuV, CLCuMuV-Raj and CLCuKoV-Sha isolates identified here encode a full length TrAP protein of 150 aa. The CLCuKoV-Bu which is the dominant strain associated with CLCuD from 2000 onward in cultivated cotton, encodes a truncated C2 protein of 35 amino acids (aa)^9, 14^. TrAP encoded by begomoviruses is a multi-functional protein, functioning as a pathogenicity determinant^44^ and also a strong suppressor of post transcriptional gene silencing activity (PTGS) of the host^45^. Full length and truncated TrAP both exhibited PTGS activity but truncated ones have lower PTGS activity as compared to full length TrAP^46^.

Recombination is a major process of evolution in begomoviruses^47, 48^. The recombination analysis shows that diversification of begomoviruses identified here occurs as a result of recombination between CLCuMuV and CLCuKoV. Most of begomoviruses identified here are recombinant between CLCuMuV and CLCuKoV exchanged as either virion-sense or complementary-sense genes but some isolates of CLCuMuV contain fragments of PeLCV and ToLCPalV in virion and complementary sense. Three types of betasatellites, replicating with begomoviruses, were identified from infected samples. A new type of recombinant betasatellite named as CLCuMuB^Veh^, has a recombinant region within the SCR region different from the previously identified CLCuMuB^Mul^ and recombinant CLCuMuB^Bur^.

The emergence of viruses associated with the first epidemic of CLCuD in cultivated cotton indicate that the begomovirus complex may be changing to resemble the original CLCuD complex found during the first epidemic of the 1990s before the introduction of resistant varieties derived from LRA5166 and CP15/2 as a source of resistance. The arrival CLCuMuV, CLCuAlV, and CLCuKoV back into cultivated cotton (Table S3) is a sign of changes in the CLCuD complex and might be a sign that a new epidemic is possible. A further study of begomovirus diversity at Vehari and surrounding areas using RCA and NGS will help to understand CLCuD in a broader sense. The changing scenario indicates the need for identification and incorporation of new sources of resistance with several recently developed technologies, into the cultivated cotton^49–51^.

## Electronic supplementary material

Supplementary Dataset 1
