# Supplementary material for: Multiple begomoviruses found associated with cotton leaf curl disease in Pakistan in early 1990 are back in cultivated cotton
Source: Sci Rep. 2017 Apr 6;7:680. doi: 10.1038/s41598-017-00727-2 (PMC5429635; doi:10.1038/s41598-017-00727-2)

-Supplementary information-

Multiple begomoviruses found associated with cotton leaf curl disease in Pakistan in early 1990 are back in cultivated cotton

Muhammad Zubair[1,2,¶], Syed Shan-e-Ali Zaidi[1,2,¶], Sara Shakir[3], Muhammad Farooq[1], Imran Amin[1], Jodi A Scheffler[4], Brian E Scheffler[5] and Shahid Mansoor[1,*]

[1]National Institute for Biotechnology and Genetic Engineering, Faisalabad, Pakistan

[2]Pakistan Institute of Engineering and Applied Sciences, Nilore, Islamabad

[3]Centre for Agricultural Biochemistry and Biotechnology, University of Agriculture, Faisalabad, Pakistan

[4]USDA-ARS, Crop Genetics Research Unit, 141 Experiment Station Rd, Stoneville, MS USA 38776

[5]USDA-ARS, Genomics and Bioinformatics Research Unit, 141 Experiment Station Rd, Stoneville, MS USA 38776

[¶]These authors contributed equally to this work.

[*] Correspondence:

Shahid Mansoor

National Institute for Biotechnology and Genetic Engineering (NIBGE), Jhang Road, Faisalabad, Pakistan,

Phone: +92-41-9201471

Fax Phone: +92-41-6201472

E-mail: Shahidmansoor7@gmail.com

Supplementary Tables

Table S1. List of different strains of begomoviruses used in RDP analysis

No	Begomoviruses
1	*African cassava mosaic virus* (ACMV)
2	*Ageratum enation virus* (AEV)
3	*Ageratum yellow vein virus* (AYVV)
4	*Bean dwarf mosaic virus* (BDMV)
5	*Bean golden mosaic virus* (BGMV)
6	*Bhendi yellow vein mosaic virus* (BYVMV)
7	*Cabbage leaf curl virus* (CabLCV)
8	*Cherry tomato leaf curl virus* (CToLCV)
9	*Chilli leaf curl virus* (ChiLCV)
10	*Cotton chlorotic spot virus* (CoChSpV)
11	*Cotton leaf crumple virus* (CLCrV)
12	*Cotton leaf curl Alabad virus* (CLCuAlV)
13	*Cotton leaf curl Bangalore virus* (CLCuBaV)
14	*Cotton leaf curl Gezira virus* (CLCuGeV)
15	*Cotton leaf curl Kokhran virus* (CLCuKoV)
16	*Cotton leaf curl Multan virus* (CLCuMuV)
17	*Cotton leaf curl Multan virus-Rajasthan* (CLCuMuV-Raj)
18	*East African cassava mosaic virus* (EACMV)
19	*Euphorbia leaf curl virus* (EuLCuV)
20	*Euphorbia yellow mosaic virus* (EuMV)
21	*Indian cassava mosaic virus* (ICMV)
22	*Malvastrum yellow vein virus* (MaYVV)
23	*Mungbean yellow mosaic India virus* (MYMIV)
24	*Mungbean yellow mosaic virus* (MYMV)
25	*Okra enation leaf curl virus* (OELCuV)
26	*Papaya leaf crumple virus* (PaLCrV)
27	*Papaya leaf curl virus* (PaLCuV)
28	*Pedilanthus leaf curl virus* (PeLCV)
29	*Pepper golden mosaic virus* (PepGMV)
30	*Pepper leaf curl virus* (PepLCV)
31	*Squash leaf curl virus* (SLCV)
32	*Sweet potato leaf curl virus* (SPLCV)
33	*Sweet potato mosaic virus* (SPMV)
34	*Tomato leaf curl Bangalore virus* (ToLCBaV)
35	*Tomato leaf curl New Delhi virus* (ToLCNDV)

Table S2. Recombination events detected by different recombination methods with their p-values by RDP4 Beta 4.74.

Isolate	Event	Recombination detection methods/ P-values								
		RDP	Geneconv	Bootscan	Max Chi Square	Chimera	Siscan	3Seq	Lard	PhylPro
MZ-1	a	2.5 e-41	6.4 e-40	5.6 e-04	7.4 e-17	3.4 e-17	6.3 e-13	--	--	2.4 e-26
	b	2.7e-50	2.9e-45	--	1.9 e-22	9.1e-24	2.1 e-40	--	--	3.9 e-61
MZ-10	c	2.5 e-41	6.5 e-40	5.62 e-04	7.37 e-17	3.4 e-17	6.3 e-13	--	--	2.4 e-26
	d	2.5 e-50	2.9 e-46	--	1.9 e-22	9.1 e-24	2.0 e-40	--	--	3.7 e-61
MZ-11	e	2.3 e-26	1.6 e-14	--	2.4 e-12	3.6 e-23	3.5 e-36	--	--	4.7 e-46
	f	1.4 e-44	1.4 e-44	--	5.2 e-29	2.5 e-30	4.1 e-31	--	--	1.7 e-76
MZ-19	h	2.3 e-26	1.6 e-14	--	2.4 e-12	3.6 e-23	3.4 e-36	--	--	4.7 e-46
	i	1.5 e-48	3.7 e-44	--	7.3 e-29	1.1 e-30	1.1 e-35	--	--	1.2 e-88
MZ-4	k	2.5 e-41	6.4 e-40	5.6 e-04	7.3 e-17	3.6 e-17	6.3 e-13	--	--	2.4 e-26
	l	2.5 e-50	2.9 e-46	--	1.9 e-22	9.1 e-24	2.1 e-40	--	--	3.7 e-61
	m	4.7 e-16	2.5 e-10	--	8.3 e-04	7.4 e-07	1.7 e-19	--	--	9.9 e-10
	n	2.5 e-50	2.9 e-46	--	1.9 e-22	9.1 e-24	2.1 e-40	--	--	3.7 e-61
MZ-52	o	3.8 e-08	1.5 e-05	--	7.1 e-05	4.2 e-03	4.5 e-07	--	--	6.4 e-03
	p	2.5 e-50	2.9 e-46	--	1.9 e-22	9.1 e-22	2.1 e-40	--	--	3.7 e-61
	q	3.8 e-08	1.5 e-05	--	7.1 e-05	4.2 e-03	4.5 e-07	--	--	6.4 e-03
	r	2.5 e-50	2.9 e-46	--	1.9 e-22	9.1 e-22	2.1 e-40	--	--	3.7 e-61

Table S3. The species and strains of cotton leaf curl viruses isolated from cotton in Pakistan. The table is based upon the dataset present at ictv.org with published and unpublished virus accessions.

Virus Species	Strain	Country	Year	Host	Abbreviation	Accession
Cotton leaf curl Alabad virus	Alabad	Pakistan	1996	unknown	CLCuAlV-Al[PK:Ala804a:96]	AJ002452
Cotton leaf curl Alabad virus	Alabad	Pakistan	1996	unknown	CLCuAlV-Al[PK:Koh802a:96]	AJ002455
Cotton leaf curl Alabad virus	Lobatum	Pakistan	2006	Lobatum	CLCuAlV-Lo[PK:Mul:Lob:06]	FJ210467
Cotton leaf curl Alabad virus	Multan	Pakistan	2006	Davidsonii	CLCuAlV-Mu[PK:Mul:Dav:06]	EU365617
Cotton leaf curl Alabad virus	Multan	Pakistan	2006	Gossypioides	CLCuAlV-Mu[PK:Mul:Gos:06]	FJ218485
Cotton leaf curl Alabad virus	Multan	Pakistan	2006	Punctatum	CLCuAlV-Mu[PK:Mul:Pun:06]	EU384575
Cotton leaf curl Kokhran virus	Burewala	Pakistan	2004	G. hirsutum	CLCuKoV-Bu[PK:Veh2:04]	AM774294
Cotton leaf curl Kokhran virus	Burewala	Pakistan	2004	G. hirsutum	CLCuKoV-Bu[PK:Kha5:04]	AM774295
Cotton leaf curl Kokhran virus	Burewala	Pakistan	2004	G. hirsutum	CLCuKoV-Bu[PK:Muz3:04]	AM774296
Cotton leaf curl Kokhran virus	Burewala	Pakistan	2004	G. hirsutum	CLCuKoV-Bu[PK:RYK7:04]	AM774297
Cotton leaf curl Kokhran virus	Burewala	Pakistan	2004	G. hirsutum	CLCuKoV-Bu[PK:DGK8:04]	AM774298
Cotton leaf curl Kokhran virus	Burewala	Pakistan	2004	G. hirsutum	CLCuKoV-Bu[PK:Fai13:04]	AM774299
Cotton leaf curl Kokhran virus	Burewala	Pakistan	2004	G. hirsutum	CLCuKoV-Bu[PK:Lod16:04]	AM774300
Cotton leaf curl Kokhran virus	Burewala	Pakistan	2004	G. hirsutum	CLCuKoV-Bu[PK:Lay20:04]	AM774301
Cotton leaf curl Kokhran virus	Burewala	Pakistan	2004	G. hirsutum	CLCuKoV-Bu[PK:Muz28:04]	AM774302
Cotton leaf curl Kokhran virus	Burewala	Pakistan	2004	G. hirsutum	CLCuKoV-Bu[PK:Ari33:04]	AM774303
Cotton leaf curl Kokhran virus	Burewala	Pakistan	2004	G. hirsutum	CLCuKoV-Bu[PK:Fai:KoB:04]	AM774304
Cotton leaf curl Kokhran virus	Burewala	Pakistan	2004	G. hirsutum	CLCuKoV-Bu[PK:Fai:KoT:04]	AM774305
Cotton leaf curl Kokhran virus	Burewala	Pakistan	2005	Xanthium strumarium	CLCuKoV-Bu[PK:Sum]	FR819707
Cotton leaf curl Kokhran virus	Burewala	Pakistan	2006	G. hirsutum	CLCuKoV-Bu[PK:Veh:06]	AM421522
Cotton leaf curl Kokhran virus	Burewala	Pakistan	2006	G. hirsutum	CLCuKoV-Bu[IN:Mul:Oct2:06]	EU365618
Cotton leaf curl Kokhran virus	Burewala	Pakistan	2006	G. hirsutum	CLCuKoV-Bu[IN:Mul:Oct5:06]	EU365619
Cotton leaf curl Kokhran virus	Burewala	Pakistan	2006	G. hirsutum	CLCuKoV-Bu[IN:Mul:Oct9:06]	EU365620
Cotton leaf curl Kokhran virus	Burewala	Pakistan	2006	G. hirsutum	CLCuKoV-Bu[IN:Mul:Oct7:06]	EU384570
Cotton leaf curl Kokhran virus	Burewala	Pakistan	2006	G. hirsutum	CLCuKoV-Bu[IN:Mul:Oct18:06]	EU384571
Cotton leaf curl Kokhran virus	Burewala	Pakistan	2006	G. hirsutum	CLCuKoV-Bu[IN:Mul:Oct20:06]	EU384572

Cotton leaf curl Kokhran virus	Burewala	Pakistan	2009	G. hirsutum	CLCuKoV-Bu[PK:Bah:09]	FR750318
Cotton leaf curl Kokhran virus	Burewala	Pakistan	2009	G. hirsutum	CLCuKoV-Bu[PK:Veh:MV2A:09]	FR750319
Cotton leaf curl Kokhran virus	Burewala	Pakistan	2009	G. hirsutum	CLCuKoV-Bu[PK:Veh:MV2B:09]	FR750320
Cotton leaf curl Kokhran virus	Burewala	Pakistan	2009	G. hirsutum	CLCuKoV-Bu[PK:RaK:MV15:09]	FR750321
Cotton leaf curl Kokhran virus	Burewala	Pakistan	2009	G. hirsutum	CLCuKoV-Bu[PK:RaK:MV16:09]	FR750322
Cotton leaf curl Kokhran virus	Burewala	Pakistan	2009	G. hirsutum	CLCuKoV-Bu[PK:Bah:MV18A:09]	FR750323
Cotton leaf curl Kokhran virus	Burewala	Pakistan	2009	G. hirsutum	CLCuKoV-Bu[PK:Bah:MV18B:09]	FR750324
Cotton leaf curl Kokhran virus	Burewala	Pakistan	2009	G. hirsutum	CLCuKoV-Bu[PK:Raj:BK1:09]	JF416947
Cotton leaf curl Kokhran virus	Burewala	Pakistan	2010	G. hirsutum	CLCuKoV-Bu[PK:Fai:C-28:10]	HF549180
Cotton leaf curl Kokhran virus	Burewala	Pakistan	2010	G. hirsutum	CLCuKoV-Bu[PK:Fai:GMT-2:10]	HF549181
Cotton leaf curl Kokhran virus	Burewala	Pakistan	2011	G. hirsutum	CLCuKoV-Bu[PK:Bha:GMT-16:11]	HF549183
Cotton leaf curl Kokhran virus	Burewala	Pakistan	2011	G. hirsutum	CLCuKoV-Bu[PK:Oka:GMT-18:11]	HF549184
Cotton leaf curl Kokhran virus	Burewala	Pakistan	2011	Luffa	CLCuKoV-Bu[PK:Bur:Luf:11]	HF567942
Cotton leaf curl Kokhran virus	Burewala	Pakistan	2012	Ricinus communis	CLCuKoV-Bu[PK:Oka:Ric:12]	HE985227
Cotton leaf curl Kokhran virus	Burewala	Pakistan	2013	G. hirsutum	CLCuKoV-Bu[PK:Cot:15]	LN845931
Cotton leaf curl Kokhran virus	Burewala	Pakistan	2013	G. hirsutum	CLCuKoV-Bu[PK:Cot:15]	LN845932
Cotton leaf curl Kokhran virus	Burewala	Pakistan	2013	G. hirsutum	CLCuKoV-Bu[PK:Cot:15]	LN713267
Cotton leaf curl Kokhran virus	Burewala	Pakistan	2013	G. hirsutum	CLCuKoV-Bu[PK:Cot:15]	LN713268
Cotton leaf curl Kokhran virus	Burewala	Pakistan	2013	G. hirsutum	CLCuKoV-Bu[PK:Cot:15]	LN713271
Cotton leaf curl Kokhran virus	Burewala	Pakistan	2013	G. hirsutum	CLCuKoV-Bu[PK:Cot:15]	LN845933
Cotton leaf curl Kokhran virus	Burewala	Pakistan	unknown	G. hirsutum	CLCuKoV-Bu[PK:For:MV13]	FR837932
Cotton leaf curl Kokhran virus	Burewala	Pakistan	unknown	G. hirsutum	CLCuKoV-Bu[PK:For:MV14]	FR837933
Cotton leaf curl Kokhran virus	Burewala	Pakistan	unknown	G. hirsutum	CLCuKoV-Bu[PK:For:MV14C]	FR837934
Cotton leaf curl Kokhran virus	Kokhran	Pakistan	1992	G. hirsutum	CLCuKoV-Ko[PK:Fai1]	AJ496286
Cotton leaf curl Kokhran virus	Kokhran	Pakistan	1995	unknown	CLCuKoV-Ko[PK:Kok72b:95]	AJ002448
Cotton leaf curl Kokhran virus	Kokhran	Pakistan	1996	unknown	CLCuKoV-Ko[PK:Man806b:96]	AJ002449
Cotton leaf curl Kokhran virus	Kokhran	Pakistan	2005	G. hirsutum	CLCuKoV-Ko[PK:Sak:05]	FN552006
Cotton leaf curl Kokhran virus	Kokhran	Pakistan	2008	Stocksii	CLCuKoV-Ko[PK:Mul:Sto1:08]	HM468427
Cotton leaf curl Kokhran virus	Layyah	Pakistan	2011	G. hirsutum	CLCuKoV-La[PK:Lay:11]	HF549182
Cotton leaf curl Kokhran virus	Shadadpur	Pakistan	2004	G. hirsutum	CLCuKoV-Sha[PK:Sha:LS4:04]	FN552004

Cotton leaf curl Kokhran virus	Shadadpur	Pakistan	2004	G. hirsutum	CLCuKoV-Sha[PK:Sha:LS6:04]	FN552005
Cotton leaf curl Kokhran virus	Shadadpur	Pakistan	2005	G. hirsutum	CLCuKoV-Sha[PK:Sha:05]	FN552001
Cotton leaf curl Kokhran virus	Shadadpur	Pakistan	2005	G. hirsutum	CLCuKoV-Sha[PK:TanJ:05]	FN552002
Cotton leaf curl Kokhran virus	Shadadpur	Pakistan	2005	G. hirsutum	CLCuKoV-Sha[PK:TanA:05]	FN552003
Cotton leaf curl Multan virus	Darwinii	Pakistan	2006	Darwinii	CLCuMuV-Dar[PK:Mul:Dar1:06]	EU365613
Cotton leaf curl Multan virus	Darwinii	Pakistan	2006	Darwinii	CLCuMuV-Dar[PK:Mul:Dar3:06]	EU365614
Cotton leaf curl Multan virus	Darwinii	Pakistan	2006	Darwinii	CLCuMuV-Dar[PK:Mul:Dar4:06]	EU365615
Cotton leaf curl Multan virus	Darwinii	Pakistan	2006	G. hirsutum	CLCuMuV-Dar[PK:Mul:Hir:06]	FJ218486
Cotton leaf curl Multan virus	Darwinii	Pakistan	2006	G. hirsutum	CLCuMuV-Dar[PK:Mul:Hir2:06]	FJ218487
Cotton leaf curl Multan virus	Faisalabad	Pakistan	1992	G. hirsutum	CLCuMuV-Fai[PK:Fai:31cf:92]	AJ496287
Cotton leaf curl Multan virus	Faisalabad	Pakistan	1992	G. hirsutum	CLCuMuV-His[PK:Fai:33af:92]	AJ496461
Cotton leaf curl Multan virus	Faisalabad	Pakistan	1995	unknown	CLCuMuV-Fai[PK:Yaz62:95]	AJ002447
Cotton leaf curl Multan virus	Faisalabad	Pakistan	1995	unknown	CLCuMuV-Fai[PK:DGK26:95]	AJ002458
Cotton leaf curl Multan virus	Faisalabad	Pakistan	1995	unknown	CLCuMuV-Fai[PK:Fai1:95]	X98995
Cotton leaf curl Multan virus	Faisalabad	Pakistan	2006	Latifolium	CLCuMuV-Fai[PK:Mul:Lat:06]	EU384573
Cotton leaf curl Multan virus	Hisar	Pakistan	1996	Okra	CLCuMuV-His[PK:Mul311:Okr:96]	AJ002459
Cotton leaf curl Multan virus	Hisar	Pakistan	1997	G. hirsutum	CLCuMuV-His[PK:Mul:H65-1:97]	AJ132430
Cotton leaf curl Multan virus	Pakistan	Pakistan	2006	Davidsonii	CLCuMuV-PK[PK:Mul:06]	EU365616
Cotton leaf curl Multan virus	Pakistan	Pakistan	2006	Mostelanum	CLCuMuV-PK[PK:Mul:Mos3:06]	EU384574
Cotton leaf curl Multan virus	Rajastahan	Pakistan	2015	G. hirsutum	CLCuMuV-Ra[PK:Veh:Cot:15]	KX656811
Cotton leaf curl Multan virus	Rajastahan	Pakistan	2015	G. hirsutum	CLCuMuV-Ra[PK:Veh:Cot:15]	KX656810
Cotton leaf curl Multan virus	Rajasthan	Pakistan	2005	Tomato	CLCuMuV-IN[PK:Fai:Tom:2005]	AM501481
Cotton leaf curl Multan virus	Rajasthan	Pakistan	2008	Digera arvensis	CLCuMuV-PK[PK:Dar:08]	FM202328
Cotton leaf curl Multan virus	Rajasthan	Pakistan	2015	G. hirsutum	CLCuMuV-Ra[PK:Veh:Cot:15]	KX656808
Cotton leaf curl Multan virus	Rajasthan	Pakistan	2015	G. hirsutum	CLCuMuV-Ra[PK:Veh:Cot:15]	KX656806
Cotton leaf curl Multan virus	Rajasthan	Pakistan	2015	G. hirsutum	CLCuMuV-Ra[PK:Veh:Cot:15]	KX656806
Cotton leaf curl Multan virus	Rajasthan	Pakistan	2015	G. hirsutum	CLCuMuV-Ra[PK:Veh:Cot:15]	KX656805
Cotton leaf curl Multan virus	Rajasthan	Pakistan	2015	G. hirsutum	CLCuMuV-Ra[PK:Veh:Cot:15]	KX656805
Cotton leaf curl Multan virus	Rajasthan	Pakistan	2015	G. hirsutum	CLCuMuV-Ra[PK:Veh:Cot:15]	KX656814
Cotton leaf curl Multan virus	Shahdadpur	Pakistan	2015	G. hirsutum	CLCuMuV-Sh[PK:Veh:Cot:15]	KX656804

Cotton leaf curl Multan virus	Shahdadpur	Pakistan	2015	G. hirsutum	CLCuMuV-Sh[PK:Veh:Cot:15]	KX656803
Cotton leaf curl Multan virus	Shahdadpur	Pakistan	2015	G. hirsutum	CLCuMuV-Sh[PK:Veh:Cot:15]	KX656802
Cotton leaf curl Multan virus		Pakistan	2015	G. hirsutum	CLCuMuV[PK:Veh:Cot:15]	KX656801
Cotton leaf curl Multan virus		Pakistan	2015	G. hirsutum	CLCuMuV[PK:Veh:Cot:15]	KX656800
Cotton leaf curl Multan virus		Pakistan	2015	G. hirsutum	CLCuMuV[PK:Veh:Cot:15]	KX656799
Cotton leaf curl Multan virus		Pakistan	2015	G. hirsutum	CLCuMuV[PK:Veh:Cot:15]	KX656797
Cotton leaf curl Multan virus		Pakistan	2015	G. hirsutum	CLCuMuV[PK:Veh:Cot:15]	KX656796
Cotton leaf curl Multan virus		Pakistan	2015	G. hirsutum	CLCuMuV[PK:Veh:Cot:15]	KX656795

Table S4. List of primers for amplification CLCuAlV

Primer name	Primer sequence
CLAlV1 F	AGAACGCACGACGATGCTC
CLAlV1 R	GAGGGACTCACCACTTAAA
CLAlV2 F	CAGAACGCACGACGATGCTC
CLAlV2 R	TGCGGGGTGTTCTTATTATCTGTC
CLAlV3 F	AAGCTGCTTCAGCTGAACCT
CLAlV3 R	TTGCCATTTGGGGACACCAT

Supplementary Figures

Figure S1: SDT analysis of *Cotton leaf curl Multan virus*-Rajastan(CLCuMuV-Raj)

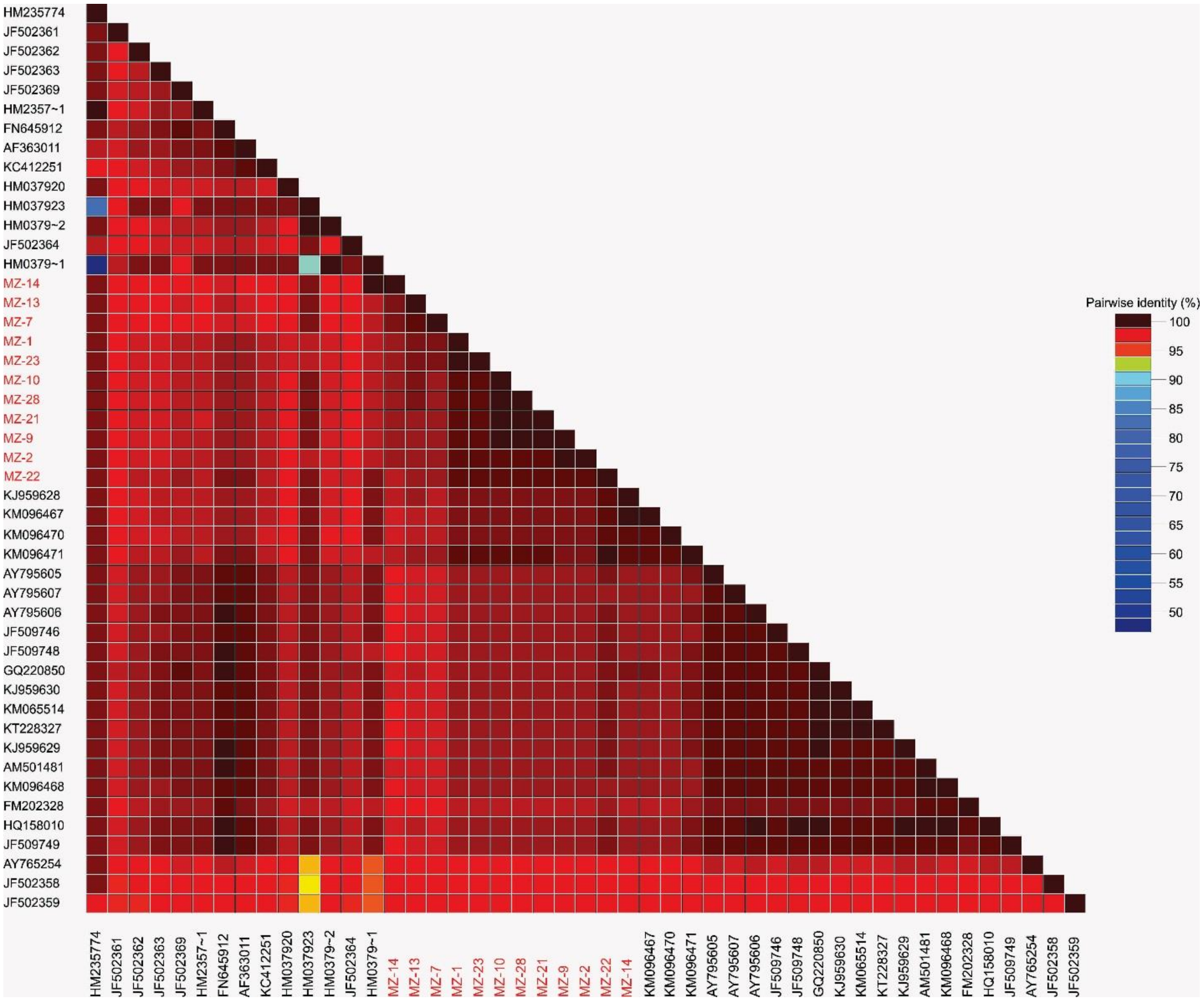

Figure S2: SDT analysis of *Cotton leaf curl Kokhran virus*-Shadaphur (CLCuKoV-Sha)

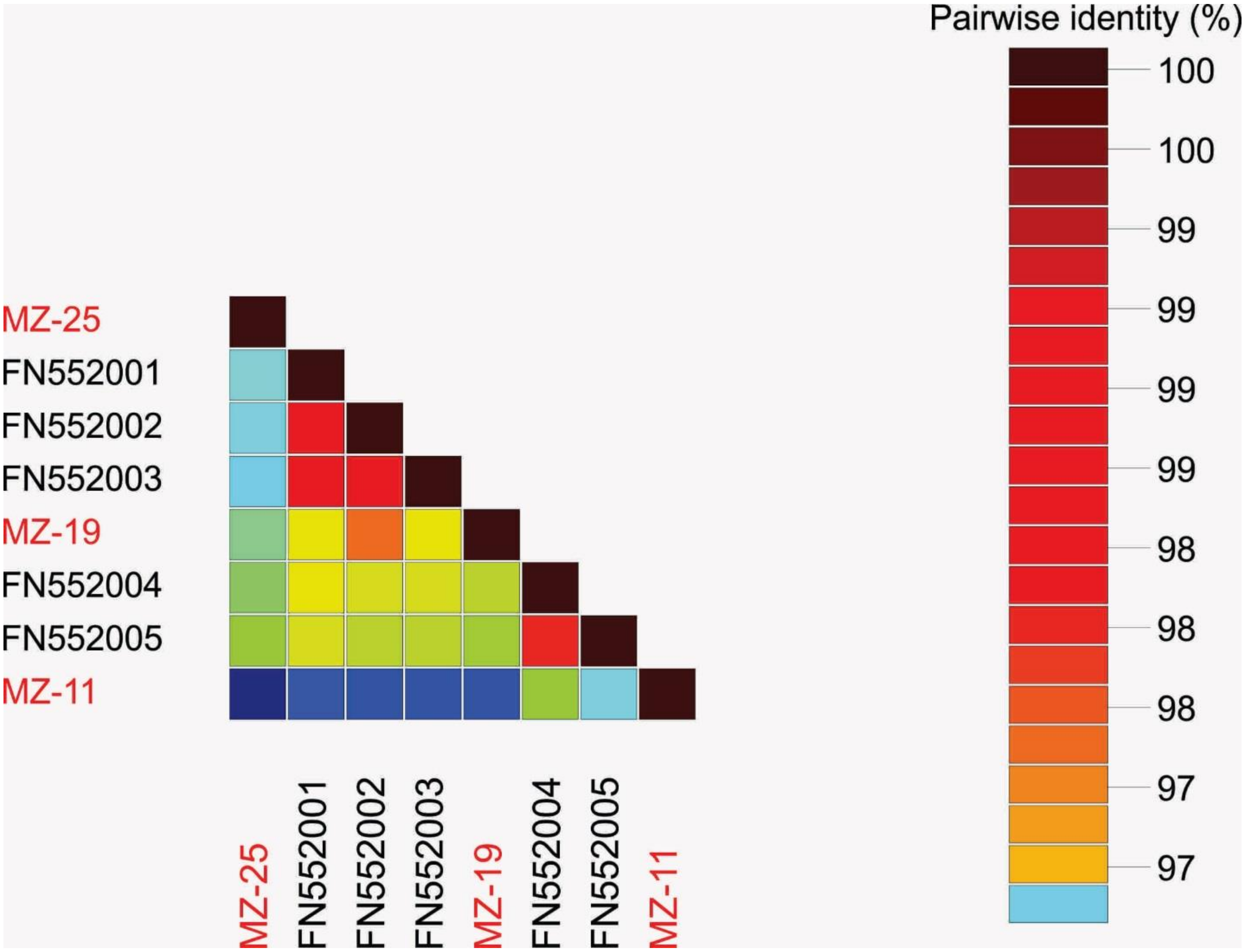

Figure S3: SDT analysis of *Cotton leaf curl Multan virus*-Pakistan (CLCuMuV-PK)

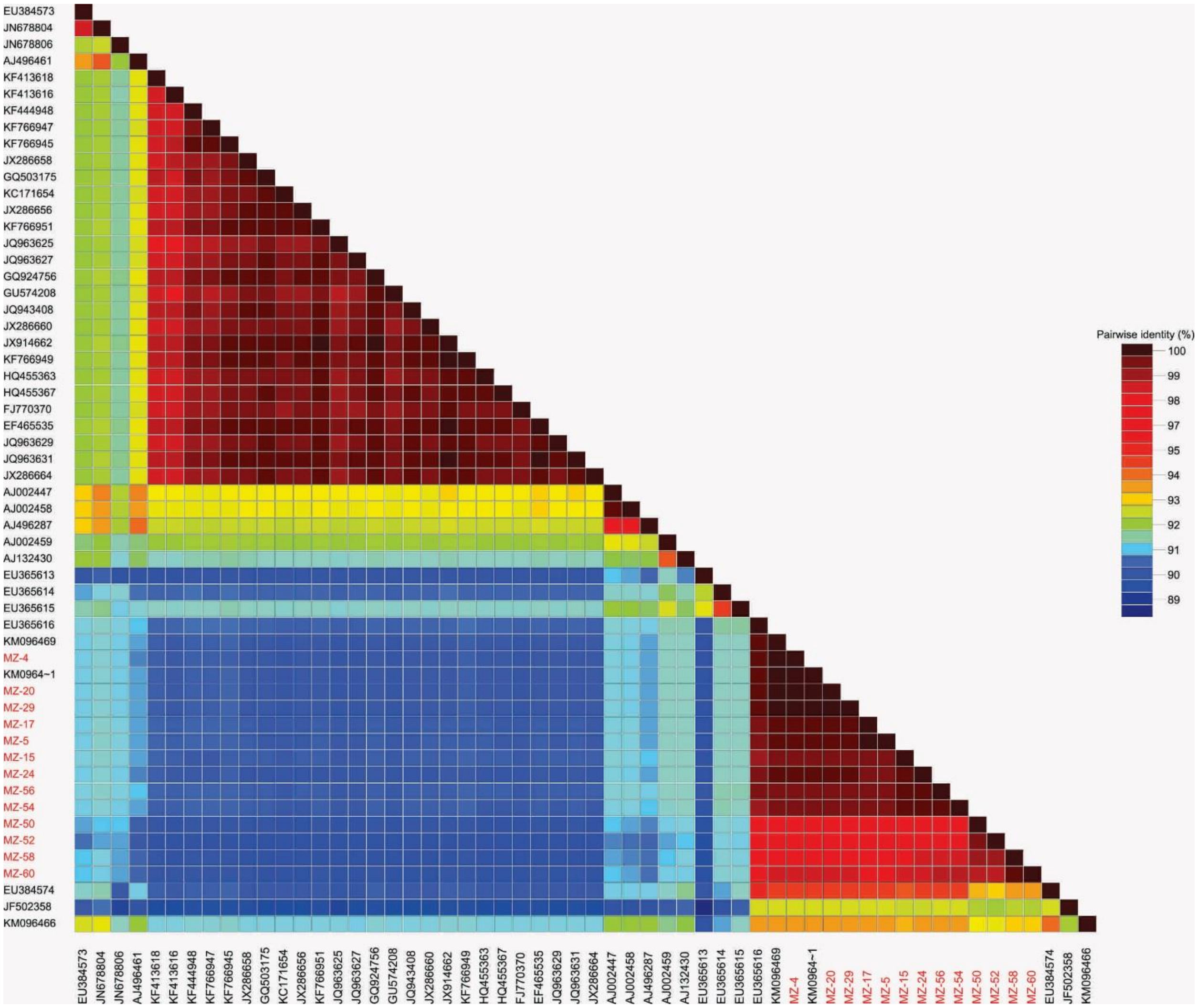

Figure S4: Recombination analysis of Ageratum conyzoides symtomless alphasatellite (AConSLA).

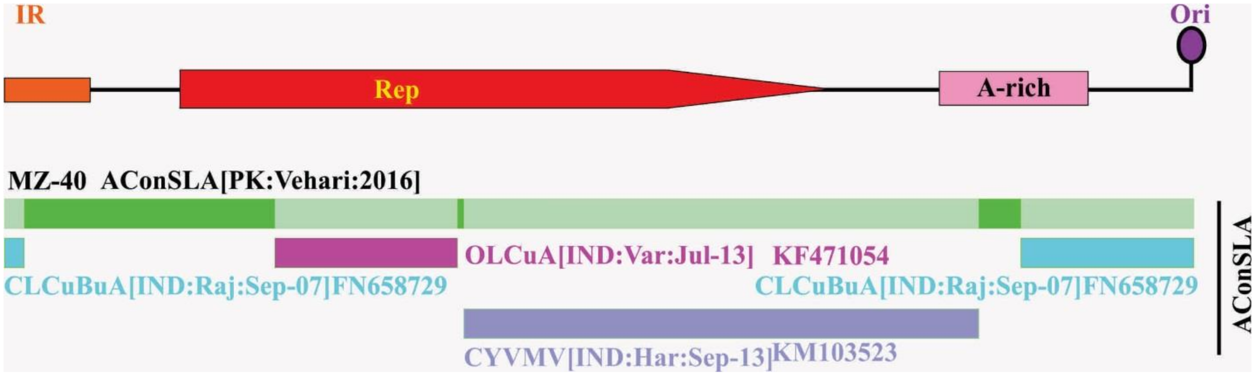

Supplement: Supplementary file 1 — Supplementary Dataset 1 [file 41598_2017_727_MOESM1_ESM.pdf]
